# Risk of lung disease in the PI*SS genotype of alpha-1 antitrypsin: an EARCO research project

**DOI:** 10.1186/s12931-024-02879-y

**Published:** 2024-06-26

**Authors:** Teresa Martín, Catarina Guimarães, Cristina Esquinas, Maria Torres-Duran, Alice M. Turner, Hanan Tanash, Carlota Rodríguez-García, Angelo Corsico, José Luis López-Campos, Eva Bartošovská, Jens-Ulrik Stæhr Jensen, José María Hernández-Pérez, Maria Sucena, Marc Miravitlles

**Affiliations:** 1 Pneumology Department, Hospital Beatriz Ângelo, Av. Carlos Teixeira 3, Loures, 2674-514 Portugal; 2 Pneumology Department, Hospital Senhora da Oliveira, Guimarães, Portugal; 3 Faculty of Medicine and Health Sciences, University of Barcelona, Barcelona, Spain; 4 Pneumology Department, NeumoVigo I+i Research Group, Hospital Álvaro Cunqueiro, IIS Galicia Sur, Vigo, Spain; 5 Respiratory Medicine, University Hospitals Birmingham NHS Foundation Trust, Birmingham, UK; 6 Institute of Applied Health Research, University of Birmingham, Birmingham, UK; 7 Department of Respiratory Medicine and Allergology, Skåne University Hospital, Lund University, Malmö, Sweden; 8 Pneumology Department, Complejo Hospitalario Clínico-Universitario de Santiago, Santiago de Compostela, Spain; 9 Pneumology Unit, IRCCS San Matteo Hospital Foundation, Pavia, Italy; 10 Department of Internal Medicine and Therapeutics, University of Pavia, Pavia, Italy; 11 Unidad Médico-Quirúrgica de Enfermedades Respiratorias, Instituto de Biomedicina de Sevilla (IBiS). Hospital Universitario Virgen del Rocío, Universidad de Sevilla, Sevilla, Spain; 12 Centro de Investigación Biomédica en Red de Enfermedades Respiratorias (CIBERES), Instituto de Salud Carlos III, Madrid, Spain; 13 Department of Pneumology, Thomayer Hospital, First Faculty of Medicine, Charles University, Prague, Czech Republic; 14 Section of Respiratory Medicine, Department of Medicine, Herlev and Gentofte Hospital, University of Copenhagen, Hellerup, Denmark; 15 Department of Clinical Medicine, Faculty of Health Sciences, University of Copenhagen, Copenhagen, Denmark; 16 Pneumology Department, Hospital Universitario Nuestra Señora de La Candelaria, Santa Cruz de Tenerife, Spain; 17 Pneumology Department, Centro Hospitalar Universitário de Santo António, Porto, Portugal; 18 Pneumology Department, Hospital Universitari Vall d’Hebron/Vall d’Hebron Research Institute (VHIR), Vall d’Hebron Barcelona Hospital Campus. CIBER de Enfermedades Respiratorias (CIBERES), Barcelona, Spain

**Keywords:** Alpha-1 antitrypsin, Lung disease, Registries, PI*SS

## Abstract

**Background:**

The PI*S variant is one of the most prevalent mutations within alpha-1 antitrypsin deficiency (AATD). The risk of developing AATD-related lung disease in individuals with the PI*SS genotype is poorly defined despite its substantial prevalence. Our study aimed to characterize this genotype and its risk for lung disease and compare it with the PI*ZZ and PI*SZ genotypes using data from the European Alpha-1 antitrypsin Deficiency Research Collaboration international registry.

**Method:**

Demographic, clinical, functional, and quality of life (QoL) parameters were assessed to compare the PI*SS characteristics with the PI*SZ and PI*ZZ controls. A propensity score with 1:3 nearest-neighbour matching was performed for the most important confounding variables.

**Results:**

The study included 1007 individuals, with PI*SS (*n* = 56; 5.6%), PI*ZZ (*n* = 578; 57.4%) and PI*SZ (*n* = 373; 37.0%). The PI*SS population consisted of 58.9% men, with a mean age of 59.2 years and a mean FEV1(% predicted) of 83.4%. Compared to PI*ZZ individuals they had less frequent lung disease (71.4% vs. 82.2%, *p* = 0.037), COPD (41.4% vs. 60%, *p* = 0.002), and emphysema (23.2% vs. 51.9%, *p* < 0.001) and better preserved lung function, fewer exacerbations, lower level of dyspnoea, and better QoL. In contrast, no significant differences were found in the prevalence of lung diseases between PI*SS and PI*SZ, or lung function parameters, exacerbations, dyspnoea, or QoL.

**Conclusions:**

We found that, as expected, the risk of lung disease associated with the PI*SS genotype is significantly lower compared with PI*ZZ, but does not differ from that observed in PI*SZ individuals, despite having higher serum AAT levels.

**Trial registration:**

www.clinicaltrials.gov (ID: NCT04180319).

## Introduction

Alpha-1 antitrypsin deficiency (AATD) is one of the most common genetic disorders in adults and is characterized by reduced levels of circulating alpha-1 antitrypsin (AAT), attributed to mutations within the *SERPINA1* gene [1]. More than 100 deficient variants have been described, being Proteinase inhibitor (PI)*S (Glu264Val) and PI*Z (Glu342Lys) the most prevalent [2]. The PI*S variant is correlated with a moderate deficiency of the protein, as it expresses 50–60% of AAT, while PI*Z is responsible for a severe deficiency, expressing 10–20% of the protein [3–5].

The most frequent clinical manifestation of AATD is pulmonary emphysema [1, 6]. The risk for developing AATD-related emphysema is well established for the PI*ZZ genotype, and an increased risk has been demonstrated for PI*SZ, particularly in smokers [7, 8]. However, it remains unclear for other genotypes, such as PI*SS. As the protein serum levels in PI*SS individuals are not below the considered protective threshold [5], the risk for pulmonary disease is believed to be low in this genotype [6]. Furthermore, PI*S is a non-polymerizing variant, with measurements of circulating polymer concentrations (with chemotactic pathogenic effects) being low in these patients [9]. However, the potential risk of lung disease associated with the PI*SS genotype may be of concern since the number of carriers of this genotype is substantial. De Serres, et al. [10]. estimated a total of 4,017,900 individuals worldwide with the PI*SS genotype, with the greatest prevalence being found in Central and Western Europe with 1,460,725 individuals, followed by 746,066 in South America and 625,651 in North Africa [10]. However, information on the burden of disease associated with the PI*SS genotype is scarce [11].

Our study aimed to characterize the PI*SS genotype and its risk for developing lung disease and compare it with the PI*ZZ and PI*SZ genotypes, using the European Alpha-1 antitrypsin Deficiency Research Collaboration (EARCO) international registry [12], which includes a large sample of individuals with the different deficient phenotypes recruited in countries in Europe and America [13].

## Method

### Study design

The EARCO registry is an international, observational, multicentre study including individuals with AATD, as detailed in the EARCO protocol [12] and registered at www.clinicaltrials.gov (ID: NCT04180319). Regarding personal data protection, the EARCO registry protocol is under the General Data Protection Regulation 2016/679 of the European Parliament and of the European Council of April 27, 2016. The EARCO database is securely hosted on the EARCO website (www.earco.org). The study protocol received central ethics approval by the Research Ethics Committee of the Vall d’Hebron University Hospital of Barcelona, Spain (PR(AG)480/2018) and was subsequently approved by all participating centres. All participants provided written informed consent and the study was conducted in accordance with the Declaration of Helsinki. The results of the baseline data of the EARCO study have recently been reported [13, 14].

The objective of the current study was to investigate the risk of lung disease associated with the PI*SS genotype by comparing the clinical characteristics and the severity of lung disease (namely, emphysema, COPD, chronic bronchitis, asthma and bronchiectasis) in subjects with the PI*SS genotype with those with the PI*SZ or PI*ZZ genotypes.

### Population

This report analyses baseline data from patients included in EARCO from February 1st, 2020, to March 1st, 2023. Patients with the PI*SS genotype were designated as the study group, while those with the PI*SZ and PI*ZZ genotypes constituted the control groups. Patients with other genotypes, those currently under augmentation therapy (AT), and those with missing information related to the genotype or AT status were excluded from the analysis.

### Measurements

We analysed the demographic data, proteinase inhibitor genotype, comorbidities, lung function, respiratory symptoms, presence of respiratory disease (including emphysema, COPD, chronic bronchitis, asthma and/or bronchiectasis) and their exacerbations. It is important to note that in the EARCO database, researchers are required to specify the type of lung disease if present, selecting from the following options: emphysema, COPD, chronic bronchitis, asthma, bronchiectasis, lung cancer or other. If “other” is chosen, researchers must provide additional details, ensuring that all types of lung disease are clearly identified and documented. We also assessed the quality of life measured by the modified medical research council (mMRC) scale, the chronic obstructive pulmonary disease (COPD) Assessment Test (CAT) specific questionnaire [15], the EuroQoL (EQ) index questionnaire [16], the EQ-Visual analogue scale (VAS), and physical activity measured by the mean time walked per day [17, 18], and the treatment administered. The severity of COPD was also measured by the body mass index, obstruction, dyspnoea and exacerbations (BODEx) index [19].

### Statistical analysis

Qualitative variables were described with absolute frequencies and percentages. The description of quantitative variables was performed using the mean and standard deviation (SD). The Kolmogorov–Smirnov test was used to assess the normality of distributions.

First, unmatched patients with the PI*SS genotype were compared with PI*ZZ and PI*SZ individuals. In the case of quantitative variables, Mann-Whitney tests were carried out. The Chi-squared test (Fisher test for frequencies < 5) was used for the comparison of categorical variables.

A propensity score matching (PSM) method was used to compare the characteristics of PI*SS with the controls, accounting for confounding variables. A 1:3 nearest-neighbour matching was performed with age, sex, pack-years, and centre as confounders, without replacement. A calliper width of 0.5 was considered at matching. Proper adjustment was assessed with standardized mean differences (SMD) in the matched population, whilst covariate imbalance was defined with an SMD threshold > 0.2.

A generalized linear model was used to evaluate the odds of Carbon monoxide transfer coefficient (KCO)(%) and forced expiratory volume in one second (FEV1)(%) being below 80% in the PI*SS group compared to PI*ZZ and PI*SZ genotypes. Generalized estimating equations were used to estimate the parameters of the generalized linear models, considering a binomial distribution and accounting for the effect raised by the clustering of patients from the same centre. A multivariable analysis was also performed, adjusting for the number of total exacerbations in the last year, and the existence of previous pneumonia. In all cases, a *p* < 0.05 was considered statistically significant. The statistical software RStudio (V2.5.1) was used for the analysis.

## Results

### Study population

The EARCO registry included 1453 individuals at the time of the analysis. A total of 446 patients were excluded for one or more of the following reasons: absence of genotype information (*n* = 30), genotype ineligible for the study (*n* = 107); or patients under AT or missing AT status (*n* = 361). The study population included 1007 individuals, with PI*SS (*n* = 56; 5.6%), PI*ZZ (*n* = 578; 57.4%) and PI*SZ (*n* = 373; 37.0%).

### Characteristics of the PI*SS population

Thirty-three (58.9%) participants were men, with a mean age of 59.2 (±13.9) years and a mean AAT level of 81 (±23.9) mg/dl. There was a predominance of former smokers (67.9%) with a mean consumption of 34.8 (±30.7) pack-years. Up to 71.4% had lung disease, COPD being the most frequent (41.1%), with a mean FEV1(%) of 83.4% (±29.9%). The mean mMRC and CAT scores were 1.69 (±0.9) and 6.5 (±7.1), respectively and the mean BODEx index was 1.22 (±1.8). The remaining characteristics are shown in Table 1.

**Table 1:** Comparison of the characteristics of PI*SS and PI*ZZ individuals in EARCO (Unmatched^a^ and Matched population^b^ (1:3))

	PI*SS (*n* = 56)	PI*ZZ (*n* = 578)^a^	*p*-value	PI*ZZ (*n* = 135)^b^	*p*-value
Demographic data:					
Age, years	59.2 (13.9)	54 (14.4)	**0.012**	56.7 (12.5)	0.474
Gender (male) (n,%)	33 (58.9%)	267 (46.2%)	0.068	71 (52.6%)	0.680
Smoking status (n,%)			**< 0.001**		0.481
Smokers Former smokers Never smokers	5 (8.9%) 38 (67.9%) 13 (23.2%)	20 (3.5%) 259 (45.1%) 295 (51.4%)		5 (3.8%) 88 (68.7%) 39 (29.5%)	
Pack-years	34.8 (30.7)	14.6 (12.9)	**< 0.001**	21.2 (17.2)	0.271
Age onset of symptoms	49.6 (19.5)	43 (15.7)	**0.010**	43 (14.3)	**0.049**
Age at diagnosis (AATD)	54.4 (16.6)	42.5 (18)	**< 0.001**	44.6 (16.7)	**0.002**
Clinical characteristics:					
Lung disease (n,%)	40 (71.4%)	427 (74.5%)	0.614	111 (82.2%)	**0.037**
COPD (n,%)	23 (41.1%)	242 (42.2%)	0.866	81 (60%)	**0.002**
Emphysema (n,%)	13 (23.2%)	245 (42.8%)	**0.005**	70 (51.9%)	**< 0.001**
Chronic bronchitis (n,%)	4 (7.1%)	25 (4.4%)	0.315	8 (5.9%)	0.737
Bronchiectasis (n,%)	10 (17.9%)	113 (19.7%)	0.737	29 (21.5%)	0.826
Asthma (n,%)	11 (19.6%)	98 (17.1%)	0.632	18 (13.3%)	0.150
Previous pneumonia (n,%)	11 (19.6%)	146 (27.3%)	0.215	42 (34.1%)	0.116
Exacerbations last 12 months, number	0.5 (1.2)	0.6 (1.2)	0.562	0.7 (0.9)	**0.050**
Charlson index (age corrected)	3.4 (2.1)	2.9 (1.9)	0.175	3.5 (1.9)	0.430
Lung function:					
FVC(%)	97.2 (22.6)	95.9 (20.9)	0.585	93.2 (23.2)	0.099
FEV1(%)	83.4 (29.9)	75.7 (29.2)	0.052	65.7 (30.3)	**< 0.001**
FEV1/FVC	0.67 (0.2)	0.62 (0.2)	0.084	0.55 (0.2)	**< 0.001**
KCO(%)	81.1 (28.5)	73.7 (22.6)	0.067	69 (22.3)	**0.001**
Blood tests:					
AAT levels	81 (23.9)	19.4 (11.2)	**< 0.001**	18.4 (11.7)	**< 0.001**
Quality of life:					
mMRC	1.69 (0.9)	1.77 [1]	0.517	2.0 (0.9)	**0.047**
CAT	6.5 (7.1)	12.3 (9.6)	**< 0.001**	15.6 (10.7)	**< 0.001**
BODEX	1.22 (1.8)	1.46 (1.9)	0.317	2.19 (2.0)	**0.001**
EQ index	0.85 (0.3)	0.84 (0.2)	0.185	0.78 (0.2)	**0.003**
EQ VAS	67.3 (27.2)	62.2 (28.4)	0.145	59.8 (25.8)	**0.026**
Physical activity (minutes walked/day)	53.1 (46.6)	56.3 (59.9)	0.898	53.7 (53.3)	0.524

Footnote: All values are mean (SD) unless otherwise specified. *P*-values obtained from the Chi-square test or the Fisher’s exact test for the categorical variables, and from the Mann-Whitney test for the continuous variables. In bold values that are statistically significant

^b^ Propensity score matching method was used to obtain the balance among baseline variables between ZZ and SS patients. To match the two cohorts, we used a 1:3 nearest-neighbour matching with age, sex, pack/year and centre, without replacement. Proper adjustment was assessed with standardized mean differences (SMD) in the matched population, whilst covariate imbalance was defined with an SMD threshold > 0.2

Abbreviations: AATD: alpha-1 antitryspin deficiency; BODEx: Body mass index, obstruction, dyspnoea and exacerbations index; COPD: chronic obstructive pulmonary disease; EQ index: EuroQol index; EQVAS: EuroQol visual analogue scale. KCO(%): Carbon monoxide transfer coefficient in percent predicted; mMRC: modified Medical Research Council

### Comparison between PI*SS and PI*ZZ. Unmatched and matched analysis

Patients in the PI*SS group were significantly older than those with PI*ZZ (59.2±13.9 vs. 54.0±14.4 years; *p* = 0.012), were less frequently never smokers (23.2% vs. 51.4%; *p* < 0.001) and had a higher smoking consumption (34.8±30.7 vs. 14.6±12.9 pack years; *p* < 0.001). However, PI*SS individuals less frequently had emphysema (23.2% vs. 42.8%; *p* = 0.005), were of an older age at symptom onset (49.6±19.4 vs. 43±15.7 years; *p* = 0.010) and had an older age at diagnosis of the deficiency (54.4±16.6 vs. 42.5±18 years; *p* < 0.001) (Table 1).

**Fig. 1:**
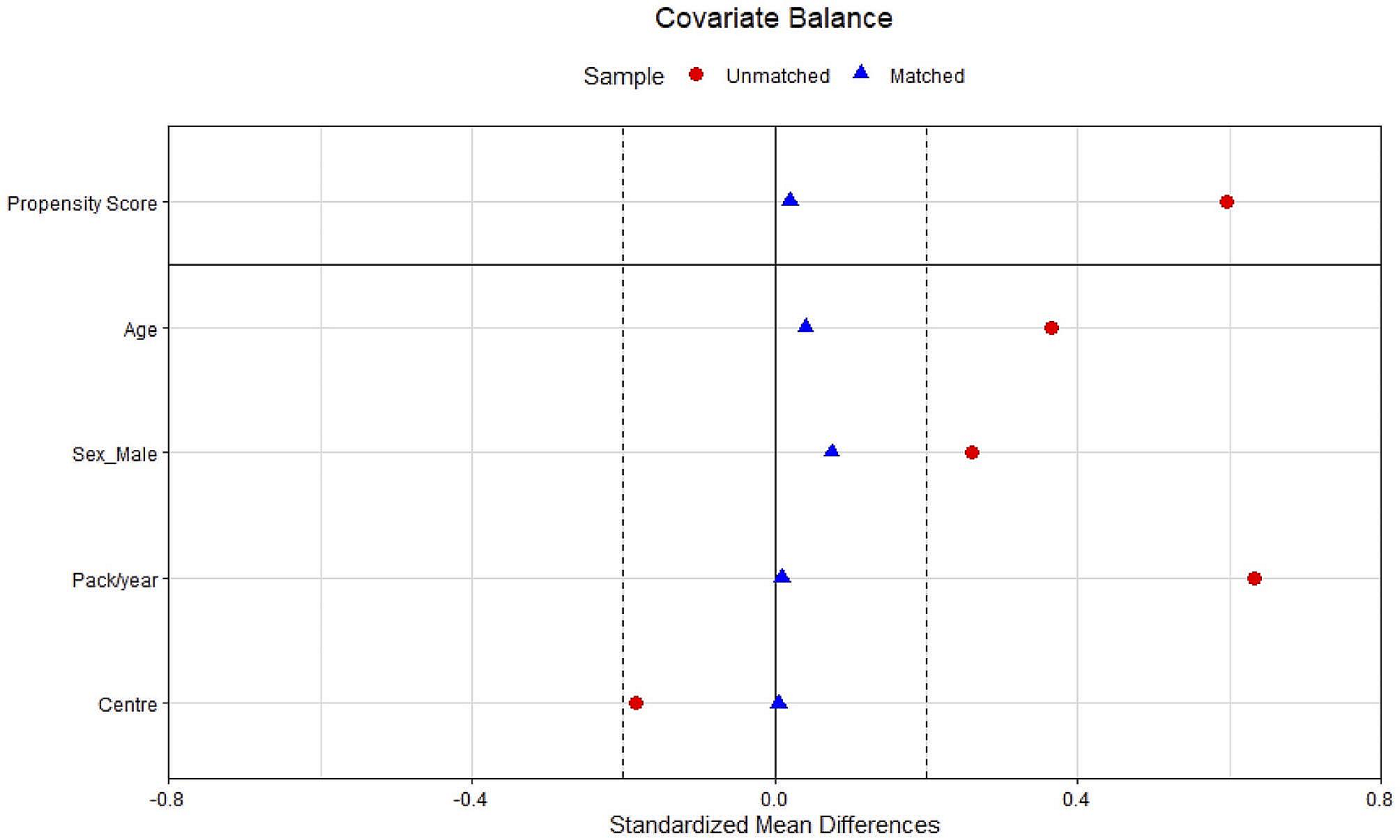
Dot plot displaying standardized mean differences in baseline characteristics between ZZ and SS patients, before (*N* = 468) and after (*N* = 185) propensity score matching Footnote: To match the two cohorts, we used a 1:3 nearest-neighbour matching with age, sex, pack-years and center, without replacement

**Fig. 2:**
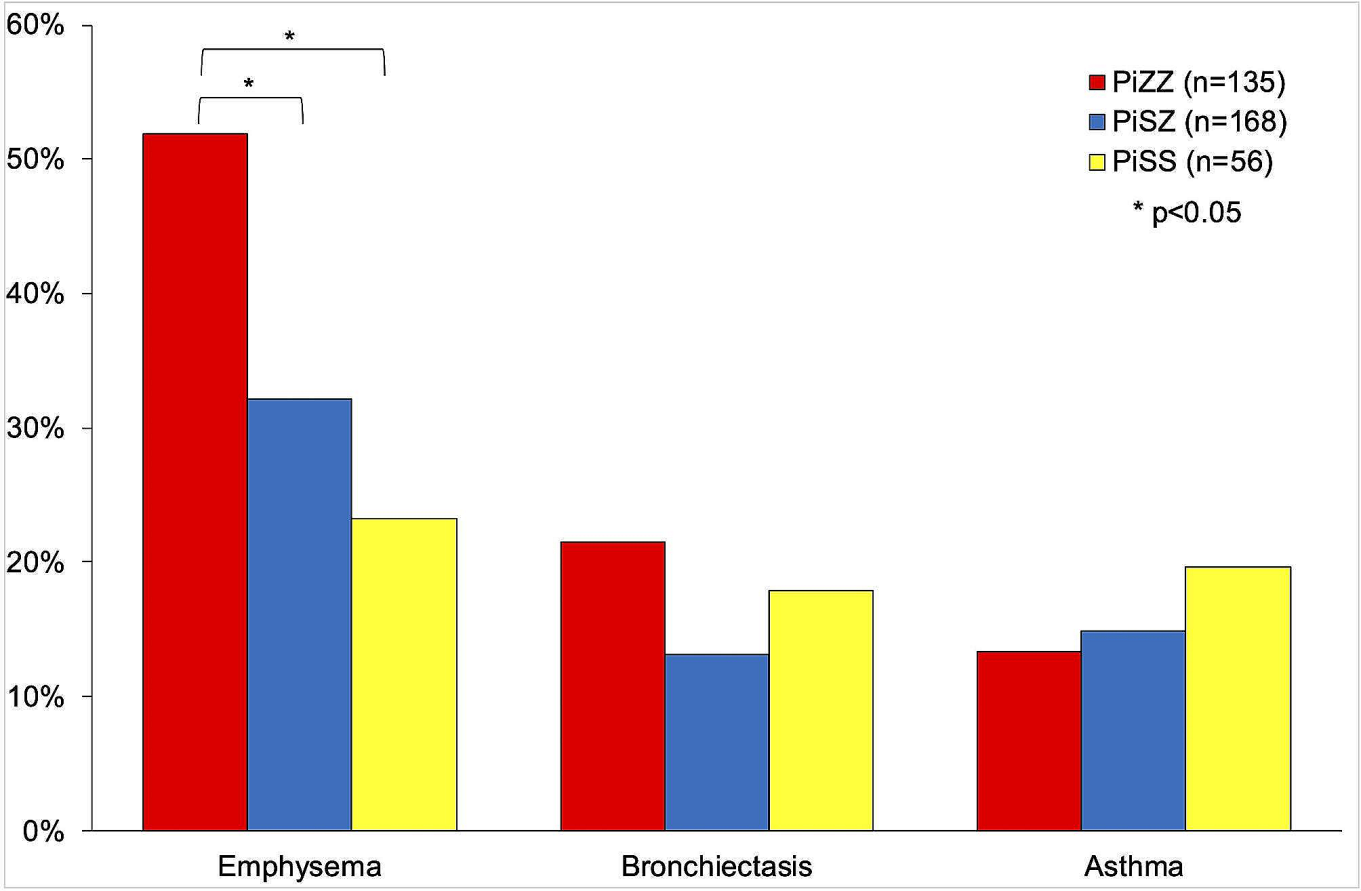
Frequency of respiratory diseases in PiZZ, PiSZ and PiSS after propensity score matching

**Fig. 3:**
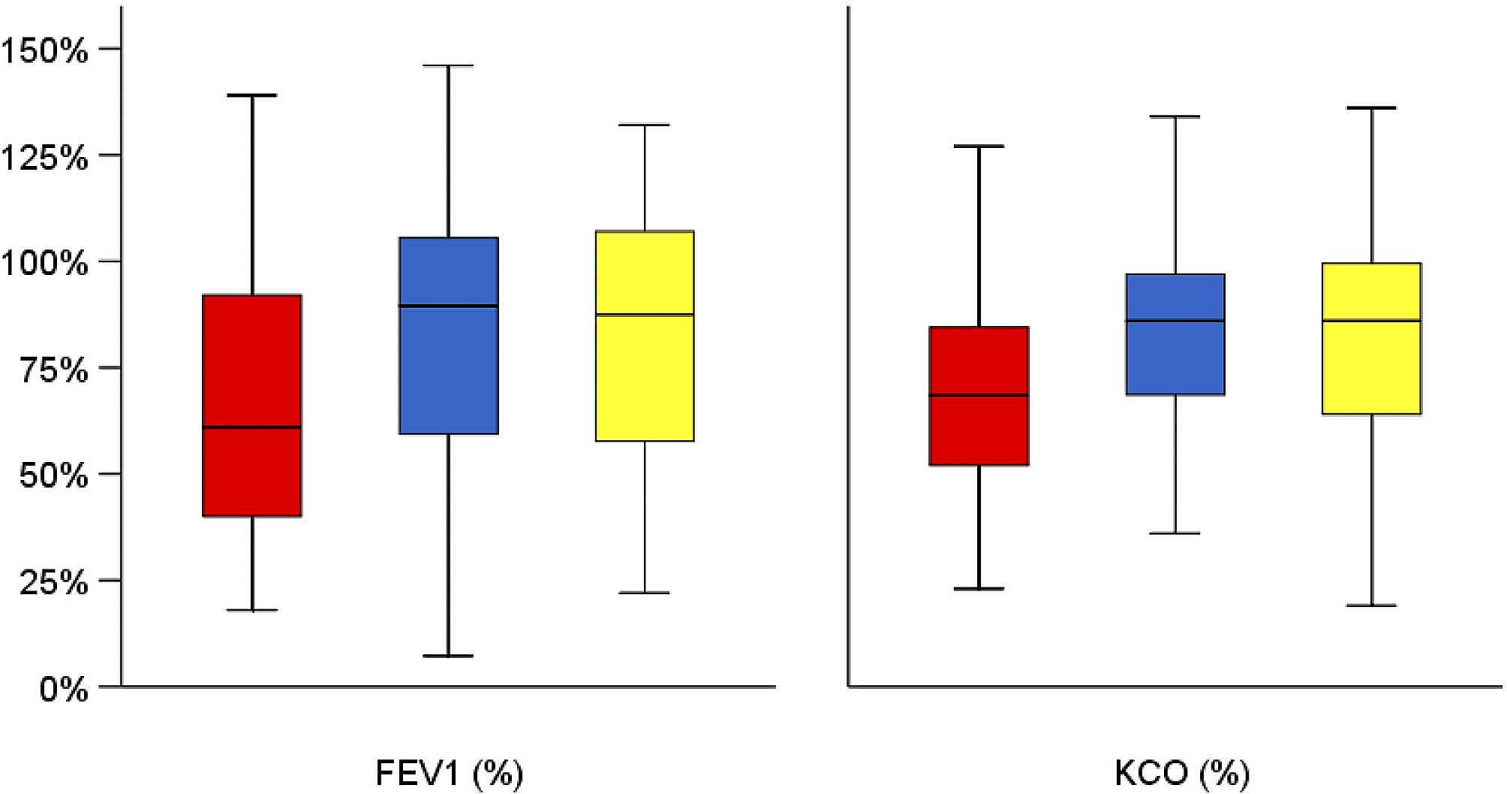
FEV1 and KCO in PiZZ, PiSZ and PiSS after propensity score matching. Footnote: *p* < 0.001 for the comparison of FEV1(%), and *p* = 0.001 for the comparison of KCO(%) between PISS and PIZZ

After matching, the characteristics of the PI*SS and PI*ZZ individuals were well balanced (Fig. 1). The matched analysis revealed similar results with a lower disease burden in individuals with PI*SS, with significant differences in lung disease (71.4% vs. 82.2%, *p* = 0.037), COPD (41.4% vs. 60%, *p* = 0.002) and emphysema (23.2% vs. 51.9%, *p* < 0.001) (Fig. 2). PI*SS individuals had an older age at diagnosis (54.4±16.6 vs. 44.6±16.7 years-old; *p* = 0.006). Lung function parameters were significantly better preserved in PI*SS subjects (Fig. 3), and they had less frequent exacerbations, a lower level of dyspnoea, a better BODEx index and better quality of life measured with the EQ-index, the VAS and CAT (Table 1).

PI*SS individuals showed a significantly lower probability of having an impaired KCO(%) (i.e. below 80% predicted) compared to PI*ZZ individuals in both the unmatched and matched cohorts and also after additional adjusting for the number of exacerbations in the last year and previous pneumonia (multivariable analysis: odds ratio [OR] 0.29, 95% confidence interval [CI]: 0.14 to 0.57; *p* < 0.001) (Table 2). In the analysis of FEV1(%), the results were similar, with an increased risk of impairment for individuals with PI*ZZ (Table 2).

**Table 2:** Generalized linear models^a^ evaluating the odds of KCO < 80% and FEV1 < 80% in PI*SS compared to PI*ZZ subjects

Variable	Univariable analysis			Multivariable analysis^b^		
	OR	95% CI	*P*-value	Adjusted OR	95% CI	*P*-value
KCO(%) < 80%						
Unmatched cohort (*N* = 634)	0.51	0.30 to 0.89	0.019	0.51	0.28 to 0.91	0.022
Matched cohort (*N* = 185)	0.30	0.15 to 0.59	0.001	0.29	0.14 to 0.57	< 0.001
FEV1(%) < 80%						
Unmatched cohort (*N* = 634)	0.69	0.40 to 1.21	0.195	0.70	0.39 to 1.23	0.242
Matched cohort (*N* = 185)	0.31	0.16 to 0.61	0.001	0.30	0.15 to 0.62	0.001

Abbreviations: FEV1(%): Forced expiratory volume in one second in percent predicted; KCO(%): Carbon monoxide transfer coefficient in percent predicted; OR: odds ratio; CI: confidence interval

^a^ Generalized estimating equations used to estimate the parameters of the generalized linear models, considering a binomial distribution and accounting for the effect raised by the clustering of patients from the same centre. ^b^ Adjusted for number of total exacerbations in the last year and previous pneumonia

### Comparison between PI*SS and PI*SZ. Unmatched and matched analysis

In the unmatched analysis, patients in the PI*SS group were older than those with PI*SZ (59.2±13.9 vs. 52.2±16.2 years; *p* = 0.002) and were less frequently never smokers (23.2% vs. 46.8%; *p* = 0.002). PI*SS subjects more frequently had lung disease (71.4% vs.- 53.9%; *p* = 0.014), particularly COPD (41.1% vs- 22.6%; *p* = 0.003) and chronic bronchitis (7.1% vs.- 1.6%; *p* = 0.030). Regarding lung function, only FEV1/FVC significantly differed, with lower values for PI*SS (0.67±0.17 vs. 0.73±0.16; *p* = 0.008). Individuals with the PI*SS genotype were more symptomatic with worse mMRC and BODEx scores (Table 3).

**Table 3:** Comparison of the characteristics of PI*SS and PI*SZ individuals in EARCO (Unmatched^a^ and Matched population^b^ (1:3))

	PI*SS (*n* = 56)	PI*SZ (*n* = 373)^a^	*p*-value	PI*SZ (*n* = 168)^b^	*p*-value
Demographics:					
Age, years	59.2 (13.9)	52.2 (16.2)	**0.002**	59.8 (13.5)	0.796
Gender (male) (n,%)	33 (58.9%)	184 (49.3%)	0.180	94 (56%)	0.697
Smoking status (n,%)			**0.002**		0.293
Smokers Former smokers Never smokers	5 (8.9%) 38 (67.9%) 13 (23.2%)	36 (9.7%) 162 (43.5%) 174 (46.8%)		17 (10.2%) 94 (56.3%) 56 (33.5%)	
Pack-years	34.8 (30.7)	26.5 (25.7)	0.080	36.9 (28.9)	0.382
Age onset of symptoms	49.6 (19.5)	45.7 (18.2)	0.147	53.5 (13.9)	0.278
Age at diagnosis (AATD)	54.4 (16.6)	46.4 (17.9)	**0.001**	53.8 (14.5)	0.600
Clinical characteristics:					
Lung disease (n,%)	40 (71.4%)	200 (53.9%)	**0.014**	111 (66.1%)	0.459
COPD (n,%)	23 (41.1%)	84 (22.6%)	**0.003**	63 (37.5%)	0.634
Emphysema (n,%)	13 (23.2%)	76 (20.5%)	0.639	54 (32.1%)	0.206
Chronic bronchitis (n,%)	4 (7.1%)	6 (1.6%)	**0.030**	4 (2.4%)	0.110
Bronchiectasis (n,%)	10 (17.9%)	36 (9.7%)	0.067	22 (13.1%)	0.376
Asthma (n,%)	11 (19.6%)	67 (18.1%)	0.775	25 (14.9%)	0.401
Previous pneumonia (n,%)	11 (19.6%)	63 (17.3%)	0.663	31 (18.8%)	0.888
Exacerbations last 12 months, number	0.52 (1.18)	0.33 (0.91)	0.248	0.43 (1.09)	0.678
Charlson index (age corrected)	3.41 (2.05)	2.62 (2.09)	**0.040**	3.4 (1.8)	0.938
Lung function:					
FVC(%)	97.2 (22.6)	101.5 (17.9)	0.262	98.3 (19.8)	0.855
FEV1(%)	83.4 (29.9)	92.7 (25.3)	0.058	83.3 (28.9)	0.893
FEV1/FVC	0.67 (0.17)	0.73 (0.16)	**0.008**	0.67 (0.18)	0.886
KCO(%)	81.1 (28.5)	87.4 (21.2)	0.478	82.9 (24.8)	0.632
Blood tests:					
AAT levels	81 (23.9)	53.9 (15.7)	**< 0.001**	54.3 (17.0)	**< 0.001**
Quality of life:					
mMRC	1.69 (0.93)	1.32 (0.92)	**0.037**	1.54 (0.98)	0.454
CAT	6.5 (7.1)	7.4 (8.4)	0.739	9 (9.6)	0.131
BODEX	1.22 (1.76)	0.64 (1.34)	**0.004**	1.07 (1.72)	0.460
EQ index	0.85 (0.26)	0.9 (0.17)	0.545	0.89 (0.17)	0.722
EQ VAS	67.3 (27.2)	73.4 (24.4)	0.185	73.2 (22.9)	0.345
Physical activity (minutes walked/day)	53.1 (46.6)	55.12 (63.9)	0.702	52.0 (62.9)	0.476

Footnote: All values are mean (SD) unless otherwise specified. *P*-values obtained from the Chi-square test or the Fisher’s exact test for the categorical variables, and from the Mann-Whitney test for the continuous variables. In bold values that are statistically significant

^b^ Propensity score matching (PSM) method was used to obtain the balance among baseline variables between ZZ and SS patients. To match the two cohorts, we used a 1:3 nearest-neighbour matching with age, sex, pack/year and centre, without replacement. Proper adjustment was assessed with standardized mean differences (SMD) in the matched population, whilst covariate imbalance was defined with an SMD threshold > 0.2

Abbreviations: AATD: alpha-1 antitryspin deficiency; BODEx: Body mass index, obstruction, dyspnoea and exacerbations index; COPD: chronic obstructive pulmonary disease; EQ index: EuroQol index; EQVAS: EuroQol visual analogue scale; KCO(%): Carbon monoxide transfer coefficient in percent predicted; mMRC: modified Medical Research Council

**Fig. 4:**
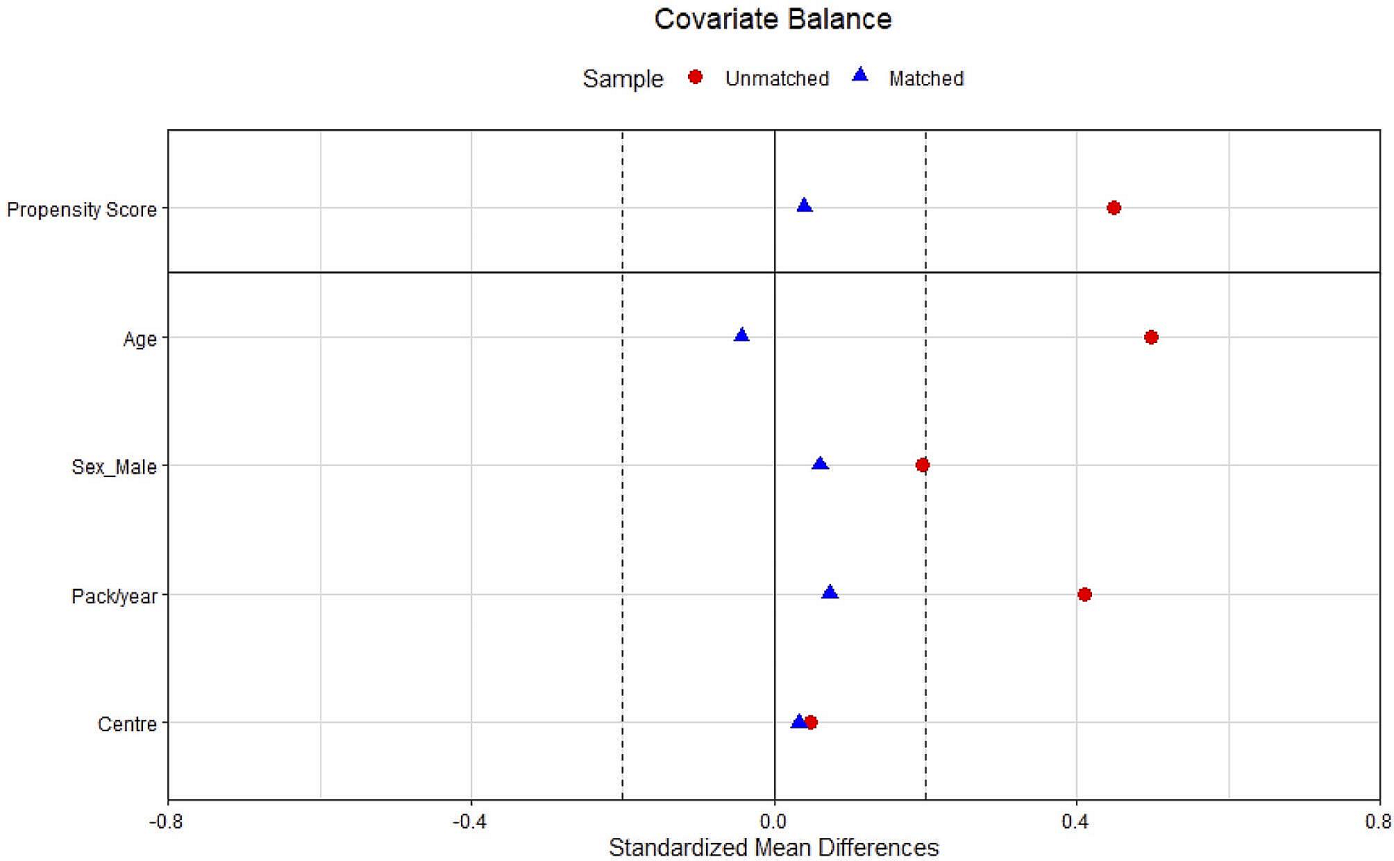
Dot plot displaying standardized mean differences in baseline characteristics between SZ and SS patients, before (*N* = 468) and after (*N* = 224) propensity score matching Footnote: To match the two cohorts, we used a 1:3 nearest-neighbour matching with age, sex, pack-years and center, without replacement

The matched analysis of PI*SS and PI*SZ individuals did not reveal significant differences in lung disease, including COPD, emphysema, bronchiectasis, and asthma (Fig. 2; Table 3). Neither were there significant differences in the age at diagnosis, the Charlson comorbidity index, lung function (Fig. 3), exacerbations, dyspnoea, quality of life indices, or physical activity. As expected, AAT levels were significantly higher in PI*SS compared to PI*SZ subjects (81±23.9 mg/dL vs. 54.3±17 mg/dL; *p* < 0.001). Figure 4 illustrates the SMD in baseline characteristics between PI*SS and PI*SZ subjects before and after propensity score matching.

Furthermore, no significant differences were found between the groups in the probability of presenting impaired KCO(%) or FEV1(%) in the matched cohort, both before and after adjusting for the additional variables of previous exacerbations and pneumonia (Table 4).

**Table 4:** Generalized linear models^a^ evaluating the odds of KCO < 80% and FEV1 < 80% in PI*SS compared to PI*SZ subjects

Variable	Univariable analysis			Multivariable analysis^b^		
	OR	95% CI	*P*-value	Adjusted OR	95% CI	*P*-value
KCO < 80%						
Unmatched cohort (*N* = 429)	1.68	0.95 to 2.98	0.075	1.59	0.89 to 2.83	0.115
Matched cohort (*N* = 224)	1.08	0.58 to 1.98	0.814	1.03	0.56 to 1.91	0.913
FEV1 < 80%						
Unmatched cohort (*N* = 429)	2.22	1.24 to 3.98	0.007	2.11	1.15 to 3.89	0.016
Matched cohort (*N* = 224)	1.10	0.60 to 2.05	0.752	1.05	0.55 to 2.02	0.875

Abbreviations: FEV1(%): Forced expiratory volume in one second in percent predicted; KCO (%): Carbon monoxide transfer coefficient in percent predicted; OR: odds ratio; CI: confidence interval

^a^ Generalised estimating equations used to estimate the parameters of the generalized linear models, considering a binomial distribution and accounting for the effect raised by the clustering of patients from the same centre. ^b^ Adjusted for number of total exacerbations last year and previous pneumonia

## Discussion

The results of our study demonstrate a decreased risk of emphysema and other lung diseases, as well as better lung function and quality of life in PI*SS individuals compared to PI*ZZ subjects in the matched population. However, to our knowledge, no direct comparisons of the risk of lung disease between PI*SS and PI*SZ have been published. Our results indicate that although there were significant differences in serum AAT levels between the PI*SS and PI*SZ genotypes, there were no significant differences in clinical manifestations, i.e. the prevalence of emphysema, COPD, asthma, bronchiectasis, or differences in lung function or quality of life, after careful matching for confounders.

The S allele (Glu264Val) is one of the most frequent variants related to AATD, causing a 40% decrease in circulating AAT protein, with mean serum levels in homozygous PI*SS subjects of 0.82 g/L (95% CI 0.73 to 1.00 g/L) [5]. In addition, the plasma neutrophil elastase inhibitory capacity of PI*SS subjects is approximately half that observed in normal PI*MM individuals, and exposure of the purified S protein to increasing oxidant burdens, as in smokers, resulted in a dose-dependent reduction in the ability of the molecule to inhibit neutrophil elastase [20]. Taken together, these findings suggest that carriers of the PI*SS genotype may be at increased risk of lung disease, particularly if they smoke. On the other hand, AAT serum levels in PI*SS subjects are usually above the considered protective threshold of 0.5 g/L, and the S protein polymerizes very slowly, resulting in negligible hepatic accumulation and circulating polymer concentrations, leading PI*S to be considered as a non-polymerizing variant [4, 9, 21], thereby suggesting a very low (or non-existent) risk of lung disease in the majority of PI*SS individuals. Clarification of the risk of lung disease associated with the PI*SS genotype would require large epidemiological studies, but there is very limited information regarding the clinical characteristics of PI*SS individuals and their risk of lung disease, particularly compared to the most frequent severe deficient genotypes PI*ZZ and PI*SZ.

For the present study we used data from the EARCO international registry, an initiative of the European Respiratory Society, aimed at collecting prospective information about patients with AATD of different deficient genotypes [12]. The EARCO registry provides a unique opportunity to investigate the characteristics of PI*SS individuals and their associated risk for lung disease. The S allele is very common in the Iberian Peninsula, with a prevalence of between 10 and 18% and is particularly frequent in Madeira Island (Portugal), with a prevalence of 18%. It is also frequent in Angola (18.8%) and Namibia (14.7%), probably related to populations of Iberian heritage [2, 4, 10].

Despite the estimated worldwide prevalence of 4,017,900 individuals with the PI*SS genotype, with the greatest prevalence in Central and Western Europe (460,725 individuals) [10], the risk of developing pulmonary disease associated with this genotype is still unclear, as most studies have focused on carriers of the PI*SZ genotype and some individuals with the PI*SS have only occasionally been described [11]. As an example, a meta-analysis on the risk of COPD due to the PI*S allele, found that the OR for COPD in PI*SZ individuals increased by 3.26 compared to normal PI*MM individuals, mainly in active and former smokers. However, there were not enough cases to summarize the risk of COPD in PI*SS homozygotes, nor was the average lung function in this genotype described due to lack of data [7]. Almost 20 years later, publications including PI*SS individuals remain very scarce.

As previously mentioned, our study found no significant differences in clinical manifestations between PI*SS and PI*SZ genotypes. Interestingly, the main lung function parameters analysed (FEV1(%) and KCO(%)) were not significantly different between PI*SS and PI*SZ individuals after matching, but in both cases they were better preserved compared to PI*ZZ subjects. Similarly, the risk of having an impaired FEV1(%) and/or KCO(%) did not significantly differ between PI*SS and PI*SZ genotypes, either before or after accounting for previous episodes of pneumonia and exacerbations. These results suggest that the antielastase protection provided by the two S alleles may be equivalent to the SZ combination; nevertheless, larger prospective studies are needed to confirm this observation.

Our findings raise important questions about the management of PI*SS individuals. Since the PI*SZ genotype increases the risk of emphysema in smokers compared to the “normal” PI*MM population [7, 8], if PI*SS individuals seem to have the same risk, counselling to quit smoking should be similarly intense for both genotypes. Furthermore, the clinical and functional monitoring of PI*SS patients who smoke should be equivalent to that carried out in PI*SZ patients.

Therapeutic considerations can also be raised, since, in selected cases, carriers of the PI*SZ genotype may be candidates for replacement therapy, as expressed in the Summary of Product Characteristics of the different approved ATs and in some guidelines [22–24]. In fact, recent data from the Spanish registry showed that 8.2% of registered PI*SZ individuals were receiving AT [25]. Again, if the risk for lung disease associated with PI*SS is not significantly different from that of PI*SZ, it may also be considered that some selected individuals with the PI*SS genotype could benefit from AT [22, 23]. However, similar to PI*SZ, there is no evidence about the efficacy of AT in this population and the great majority (if not all) of subjects with the PI*SS genotype have serum levels above those considered to be protective [5, 6]. In any case, if our results showing a similar risk of lung disease for PI*SS and PI*SZ are confirmed in larger series, this could justify the inclusion of PI*SS subjects in clinical trials of other future preventive treatments under development, such as oral or inhaled elastase inhibitors. It should also encourage debate as to whether there is a universal protective AAT level, and if so where it lies.

The S allele has also been associated with a higher prevalence and increased severity of asthma. Data from the Spanish registry, one of the largest reported samples of PI*SZ individuals, showed that the prevalence of asthma was significantly higher for PI*SZ compared to PI*ZZ subjects [25]. Recent studies have also observed that carriers of the S allele may have more frequent and severe asthma exacerbations [26, 27]. However, our study did not find any significant difference in the prevalence of asthma among the three groups. In fact, the prevalence of neither asthma nor bronchiectasis showed significant differences among the PI*SS, PI*SZ and PI*ZZ genotypes after matching. In any case, our data cannot rule out a possible influence of the S allele on the clinical manifestations, severity or exacerbations of asthma.

Our study has some limitations: firstly, despite careful matching for risk factors we cannot rule out the possibility of other confounders that were not considered. Secondly, there may be some selection bias derived from the potential tendency to register in EARCO the most affected PI*SS individuals, who are the those most frequently diagnosed, but this bias may also apply for the PI*SZ and PI*ZZ individuals. Thirdly, we could not compare PI*SS with a matched cohort of normal PI*MM individuals and, therefore, we can only speculate about an increased risk of lung disease in the PI*SS versus the normal population based on an indirect comparison through the similar risk observed with the PI*SZ genotype. Fourthly, it should be noted that patients in the group characterized by the PI*SS genotype represent only 5.6% of the entire deficient population analysed. Finally, we do not yet have prospective follow-up data that could provide insights into the risk of exacerbations and lung function decline associated with the PI*SS genotype. Future larger studies and/or the future analysis of prospective EARCO data may provide more information about the evolution of these subjects. Conversely, this is the largest study characterizing individuals with the PI*SS genotype and the comparison of these subjects not only with PI*ZZ, but perhaps more importantly, with participants with the PI*SZ genotype, a known risk factor for lung disease in smokers [7, 8] and using propensity score matching and a multivariate analysis of risk factors.

## Conclusion

The results of our study suggest that subjects with the PI*SS genotype have a significantly lower risk of lung disease compared with the PI*ZZ genotype, but after careful matching accounting for the most important known confounders we did not find evidence for any significant difference in the risk of lung disease between the PI*SS and PI*SZ genotypes. Since the PI*SZ genotype is considered a genetic risk factor for the development of lung disease in smokers, careful attention and strong anti-smoking counselling should also be given to PI*SS subjects that smoke.

## Data Availability

No datasets were generated or analysed during the current study.

## Abbreviations

AAT: Alpha-1 antitrypsin
AATD: Alpha-1 antitrypsin deficiency
BODEx: Body mass index, obstruction, dyspnoea, and exacerbations index
CAT: Chronic obstructive pulmonary disease (COPD) Assessment Test
COPD: Chronic Obstructive Pulmonary Disease
EARCO: European Alpha-1 antitrypsin Deficiency Research Collaboration
EQ: Euro-Quality of life index questionnaire
FEV1: Forced expiratory volume in one second
FVC: Forced vital capacity
KCO: Carbon monoxide transfer coefficient
mMRC: Modified Medical Research Council
PI: Proteinase inhibitor
QoL: Quality of life
PSM: Propensity score matching
SD: Standard deviation
SMD: Standardized mean differences
VAS: Visual analogue scale
